# *De novo* non-synonymous TBL1XR1 mutation alters Wnt signaling activity

**DOI:** 10.1038/s41598-017-02792-z

**Published:** 2017-06-06

**Authors:** Akira Nishi, Shusuke Numata, Atsushi Tajima, Xiaolei Zhu, Koki Ito, Atsushi Saito, Yusuke Kato, Makoto Kinoshita, Shinji Shimodera, Shinji Ono, Shinichiro Ochi, Akira Imamura, Naohiro Kurotaki, Shu-ichi Ueno, Nakao Iwata, Kiyoshi Fukui, Issei Imoto, Atsushi Kamiya, Tetsuro Ohmori

**Affiliations:** 1 Department of Psychiatry, Graduate School of Biomedical Sciences, Tokushima University, Tokushima, Japan; 2 Department of Bioinformatics and Genomics, Graduate School of Advanced Preventive Medical Sciences, Kanazawa University, Ishikawa, Japan; 3 Department of Human Genetics, Graduate School of Biomedical Sciences, Tokushima University, Tokushima, Japan; 4 Department of Psychiatry and Behavioral Sciences, Johns Hopkins University School of Medicine, Baltimore, MD USA; 5 Division of Enzyme Pathophysiology, The Institute for Enzyme Research (KOSOKEN), Tokushima University, Tokushima, Japan; 6 Department of Neuropsychiatry, Kochi Medical School, Kochi University, Kochi, Japan; 7 Department of Neuropsychiatry, Nagasaki University Graduate School of Biomedical Sciences, Nagasaki, Japan; 8 Department of Neuropsychiatry, Ehime University Graduate School of Medicine, Ehime, Japan; 9 Department of Psychiatry, School of Medicine, Fujita Health University, Toyoake, Aichi Japan

## Abstract

Here we report *de novo* non-synonymous single-nucleotide variants (SNVs) by conducting whole exome sequencing of 18 trios consisting of Japanese patients with sporadic schizophrenia and their parents. Among nine SNVs, we explored the functional impact of the *de novo* mutation in *TBL1XR1* [c.30 C > G (p.Phe10Leu)], a gene previously found to be associated with autism spectrum disorder and epilepsy. Protein structural analysis revealed that Phe10Leu mutation may decrease the structural stability of the TBL1XR1 protein. We demonstrate that Phe10Leu mutation alters the interaction of TBL1XR1 with N-CoR and β-catenin, which play critical roles in regulation of Wnt-mediated transcriptional activity. Consistently, TBL1XR1-mediated activation of Wnt signaling was up-regulated by Phe10Leu mutation. These results suggest that a *de novo* TBL1XR1 point mutation could alter Wnt/β-catenin signaling activity. Further studies are required to clarify the involvement of TBL1XR1 mutations in neuropsychiatric conditions.

## Introduction

Schizophrenia is a complex condition resulting from genetic and environmental etiological influences^1^. A meta-analysis of twin studies revealed that the point estimate of heritability of schizophrenia is 81%^2^. To date, multiple common and rare genetic variants have been identified as genetic risk factors for schizophrenia^3–5^, while there are a number of patients with no family history of schizophrenia, so called sporadic cases.

Lynch (2010) estimated that the average newborn acquires a total of 50–100 new mutations, resulting in approximately 0.86 novel amino acid-altering mutations per generation^6^. *De novo* mutations, such as single-nucleotide variants (SNVs), insertions and deletions (INDELs), and copy-number variants (CNVs) may contribute to the genetic etiology of sporadic schizophrenia and may explain the high prevalence rate of schizophrenia in general population. Recent trio-based studies using next-generation sequencing technology have identified *de novo* SNVs in sporadic schizophrenia^7–13^. Nonetheless, these rare SNVs are uncommon across studies, and molecular mechanisms of these mutations underlying schizophrenia still remain obscure. No whole-exome sequencing studies of sporadic schizophrenia have been previously reported in the Japanese population.

In the present study, we conducted whole exome sequencing of 18 trios consisting of patients with sporadic schizophrenia and their parents, and we identified *de novo* non-synonymous SNVs. We further examined the effect of the novel *de novo TBL1XR1* mutation [c.30 C > G (p.Phe10Leu)] on the protein structure of TBL1XR1 and Wnt/β-catenin signaling pathway.

## Results

### *De novo* SNVs identified in schizophrenia trio samples by exome sequencing

We conducted exome sequencing of 18 trios. On average, we obtained 15.4 GB of raw sequence data per sample, and 96.9% of these data were mapped to the reference genome (hg19). On detection of *de novo* mutations, we found 82 *de novo* SNVs in 18 trios. Of these 82 *de novo* SNVs, 17 were predicted to be non-synonymous mutations. Of these 17 *de novo* non-synonymous SNVs, we validated nine mutations in eight trios by Sanger sequencing (Table 1). Among these nine *de novo* non-synonymous SNVs, two mutations, one in the *ABCD4* [ATP-binding cassette, sub-family D (ALD), member 4] gene and one in the *TBL1XR1* [transducin (beta)-like 1 X-linked receptor 1] gene, were predicted as damaged by all of three software tools (Table 1). Given that *de novo TBL1XR1* point mutations have been found in other neuropsychiatric conditions, including autism spectrum disorder (ASD) and West syndrome^14, 15^, we conducted subsequent structural and functional analyses of the observed *de novo* non-synonymous *TBL1XR1* mutation [c.30 C > G (p.Phe10Leu)]. The *TBL1XR1* point mutation (p.Phe10Leu) was not observed either in the independent 1,191 patients with schizophrenia nor in the 1,986 non-psychiatric control subjects.

**Table 1:** *De novo* non-synonymous missense mutations.

Trio ID	Sex^a^	Gene	Chr^b^	Position	Nucleotide change (Ref > Obs)^c^	Amino acid change	PolyPhen-2^d^	SIFT	PROVEAN
4	F	SUPT6H	17	27028608	G > A	p.Asp1716Asn	probably	Damaging	Neutral
6	F	KRT34	17	39538037	G > A	p.Ala162Val	benign	Damaging	Neutral
7	F	SLTM	15	59179560	T > C	p.Glu421Gly	probably	Damaging	Neutral
8	M	TBL1XR1	3	176782736	G > C	p.Phe10Leu	possibly	Damaging	Deleterious
9	M	POLR3F	20	18455749	G > C	p.Ser116Thr	possibly	Tolerated	Neutral
12	F	FARS2	6	5431283	T > C	p.Ile261Thr	benign	Tolerated	Neutral
12	F	LEMD3	12	65633735	C > T	p.Arg650Cys	benign	Tolerated	Neutral
13	F	ABCD4	14	74759077	A > G	p.Ile344Thr	possibly	Damaging	Deleterious
15	M	DNAJA1	9	33036612	G > C	p.Val267Leu	possibly	Tolerated	Neutral

Using hg19 as the human reference genome.

^a^Sex; M = Male, F = Female.

^b^Chr = Chromosome.

^c^Ref = reference genome sequence, Obs = observed geneme sequence.

^d^PolyPhen-2; probably = probably damaging, possibly = possibly damaging.

Degree of damaging “probably” > “possibly” > “benign”.

### Phe10Leu mutation impairs structural stability of TBL1XR1 protein

**Figure 1:**
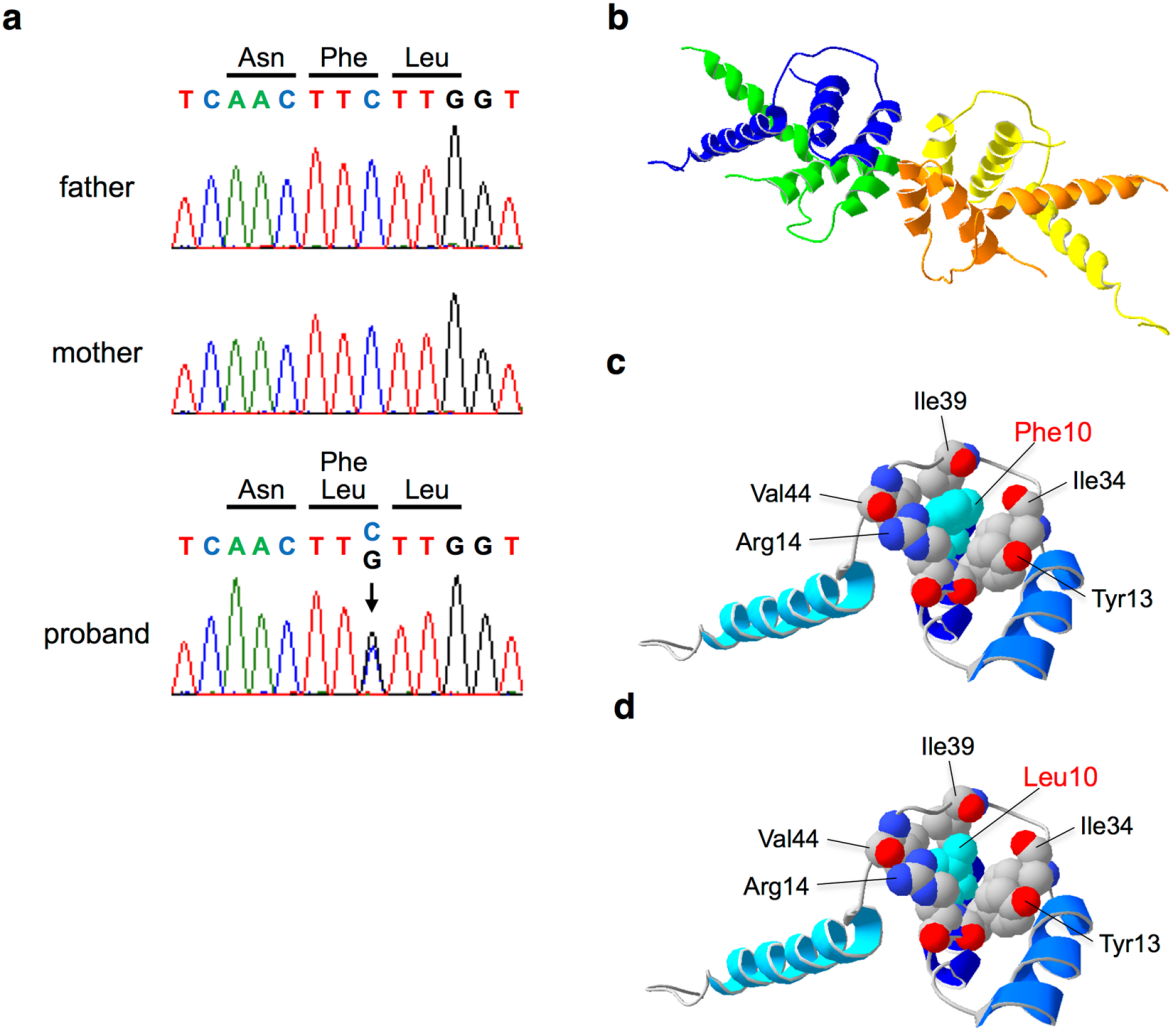
Structural models of TBL1XR1 N-terminal domain (NTD). (**a**) *De novo* mutation in TBL1XR1 [c.30 C > G (p.Phe10Leu)]. The chromatogram shows the mutation in the TBL1XR1 gene, which is observed in the proband (arrow) but not in the parents. (**b**) Overall structure of a homology model of tetrameric NTD of TBL1XR1. Monomers are depicted in distinct colors. (**c**) A monomer model of the control NTD is depicted as a ribbon model. Important residues are depicted as spheres. Gray, red and blue spheres indicate carbon, oxygen and nitrogen atoms, respectively, although all atoms of Phe10 are colored in cyan for clarity. (**d**) A monomer model of Phe10Leu NTD is depicted as a ribbon model. Leu10 is colored in cyan for clarity.

We next examined the effect of the *TBL1XR1* point mutation (p.Phe10Leu) on the protein structure of TBL1XR1 (Fig. 1a). Surface areas of Phe and Leu amino acids were 175 and 137 Å^2^, respectively (Table 2a). Van der Waals (VdW) volumes of Phe and Leu were 135 and 124 Å^2^, respectively. Thus, we predict that substitution of Leu for Phe may decrease the volume and surface area of the 10th residue of TBL1XR1. TBL1XR1 is composed of two structural domains, the N-terminal domain (NTD) and tryptophan-aspartic acid 40 (WD40) repeat. The Phe10Leu mutation is located within the NTD. To assess the substitution in the context of protein structure, we built structural models of the NTD of the control and Phe10Leu TBL1XR1, and we obtained tetramer models of the NTD (Fig. 1b). In a monomer model of the control NTD, the side chain of Phe10 interacts with those of Tyr13, Arg14, Ile34, Ile39, and Val44 within the monomer (Fig. 1c). In the Phe10Leu model, the region around the 10th residue appears sparser than the control (Fig. 1d). We therefore calculated contact areas of the surrounding residues with the rest of the structures and found that those of the surrounding residues, except for Val44, were decreased in the Phe10Leu mutant (Table 2b). Calculated stability of protein structures was lower in the Phe10Leu NTD model than in the control (Table 2c). It is remarkable that statistics associated with VdW potentials were higher in the Phe10Leu NTD. Collectively, the results suggest that the Phe10Leu substitution of NTD decreases structural stability due to the decreases in the contact area of the 10th residue with the surrounding residues.

**Table 2:** Results of structural analysis.

		VdW volume (Å^3^)		surface area (Å^2^)	
**a. Volume and surface area of Phe and Leu**					
Phe		135		175	
Leu		124		137	
	**Tyr13**	**Arg14**	**Ile34**	**Ile39**	**Val44**
**b. Buried surface area of important residues**					
Control	10.1	14.6	16.7	24.8	5.3
Phe10Leu	4.0	9.7	11.9	22.3	7.8
	**Control**	**Phe10Leu**		**Difference**	
**c. Statistics for structural potentials**					
BackHbond	−53.66	−54.02		−0.36	
SideHbond	−5.87	−5.27		0.6	
Energy_VdW	−59.41	−58.84		0.57	
Electro	−0.7	−0.75		−0.05	
Energy_SolvP	90.93	90.44		−0.49	
Energy_SolvH	−71.61	−71.14		0.47	
Energy_VdWclash	8.17	9.19		1.02	
energy_torsion	0.75	1.22		0.47	
backbone_VdWclash	64.86	64.69		−0.17	
Entropy_sidec	26.84	27.06		0.22	
Entropy_mainc	97.74	97.14		−0.6	
water bonds	0	0		0	
helix dipole	−0.13	−0.13		0	
loop_entropy	0	0		0	
cis_bond	0	0		0	
disulfide	0	0		0	
kn electrostatic	0	0		0	
partial covalent interactions	0	0		0	
Energy_Ionisation	0.1	0.1		0	
Entropy Complex	0	0		0	
Total	33.16	35		1.84	

### Phe10Leu mutation of TBL1XR1 alters Wnt signaling activity

**Figure 2:**
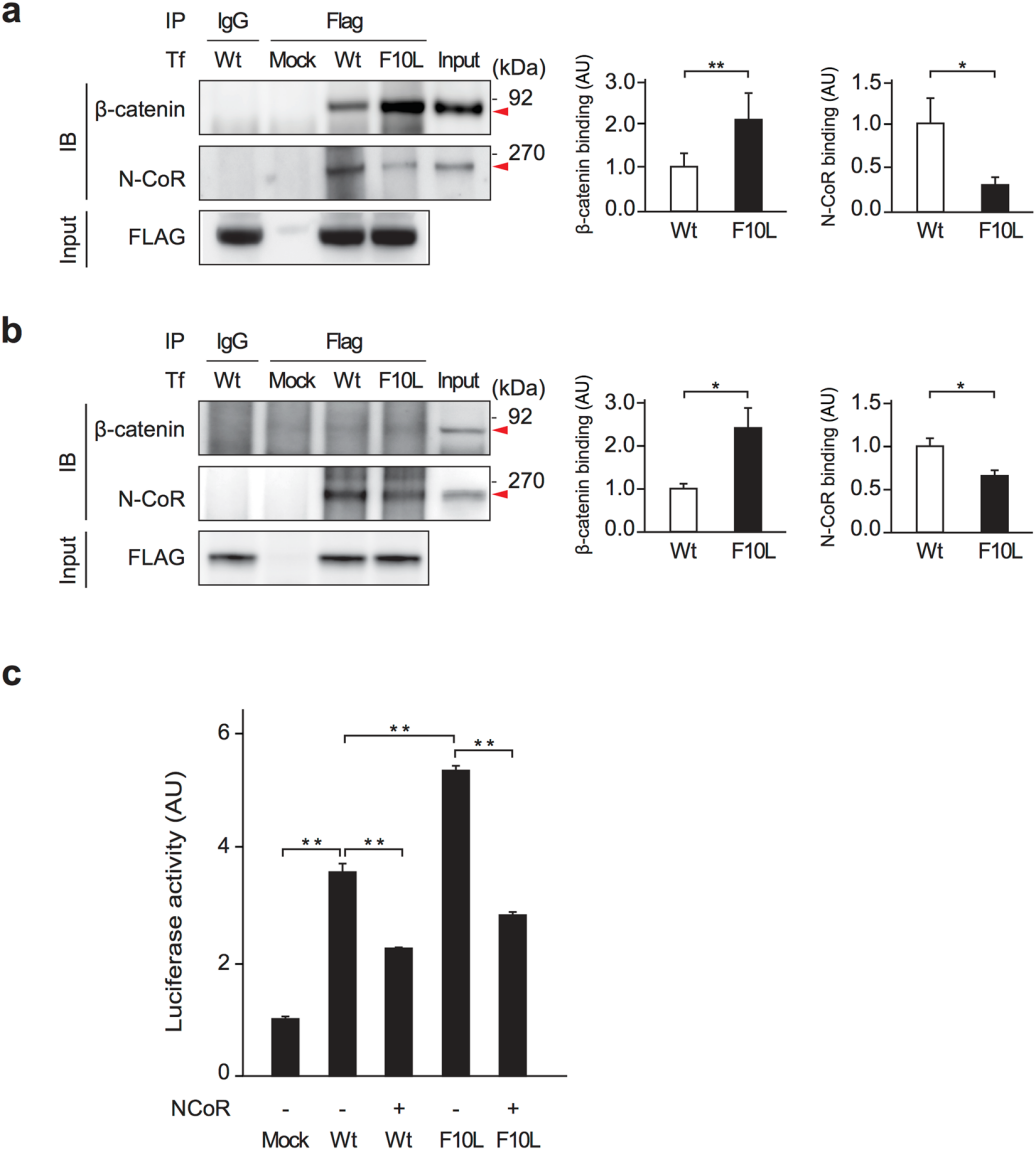
The effect of the Phe10Leu mutation (F10L) on the protein interaction of TBL1XR1 with N-CoR and β-catenin as well as Wnt/β-catenin transcription activity. (**a,b**) Interaction of wild-type and F10L mutant TBL1XR1 (TBL1XR1^Phe10Leu^) with endogenous N-CoR and β-catenin was assessed in 293FT cells and HT22 hippocampal neuronal cells by co-immunoprecipitation experiments. TBL1XR1^Phe10Leu^ displays stronger binding with β-catenin compared to wild-type TBL1XR1 (red arrowhead in top panel), while the binding of TBL1R1^Phe10Leu^ and N-CoR is weaker than that of wild-type TBL1XR1 (red arrowhead in middle panel) (*P < 0.05 and **P < 0.01). The inputs of each protein are also shown (bottom panel). Full immunoblots are presented in Supplementary Figure. (**c**) The TOPFlash Wnt reporter assay showed that overexpression of wild-type TBL1XR1 increased Wnt transcription activity, which was further enhanced by an F10L mutation in TBL1XR1 (*P < 0.05 and **P < 0.01). Overexpression of N-CoR suppresses an increase in Wnt transcriptional activity induced by either wild-type TBL1XR1 or F10L mutant (**P < 0.01). Luciferase activities were determined 48 hours post-transfection and normalized against Renilla values. Bars represent averages of each group in three independent experiments. AU, arbitrary unit. All data are presented as the mean ± s.e.m.

TBL1XR1 is a component of the nuclear receptor corepressor (N-CoR) and silencing mediator of retinoic acid and thyroid hormone receptor (SMRT) protein complex, which regulates transcriptional repression machinery^16^. TBL1XR1 also plays a critical role in recruiting β-catenin to Wnt target gene promoters for transcription activation^17^. To examine whether the Phe10Leu mutation affects TBL1XR1-mediated transcription mechanisms, either wild-type TBL1XR1 or the Phe10Leu mutant (TBL1XR1^Phe10Leu^) was exogenously expressed in 293T cells and co-immunoprecipitated with N-CoR and β-catenin. While the Phe10Leu mutation disturbed the interaction between TBL1XR1 and N-CoR, we observed increased binding of TBL1XR1 to β-catenin (Fig. 2a). Consistently, reduced binding of TBL1XR1 to N-CoR and increased interaction between TBL1XR1 and β-catenin were observed in HT22 cells, mouse hippocampal neuronal cells overexpressing TBL1XR1^Phe10Leu^ (Fig. 2b). Next, we examined the effect of the Phe10Leu mutation on Wnt/β-catenin-mediated transcriptional activity by using the TOPFlash Wnt reporter assay^18^. Consistent with previous studies^17, 19^, overexpression of wild-type TBL1XR1 enhanced Wnt transcriptional activity, which was further up-regulated by Phe10Leu mutation (Fig. 2c). Interestingly, although Phe10Leu mutation decreases the interaction between TBL1XR1 and N-CoR, overexpression of N-CoR suppresses enhancement of Wnt transcriptional activity induced by either wild-type TBL1XR1 or Phe10Leu mutant (Fig. 2c).

## Discussion

To the best of our knowledge, this is the first study conducting trio-based exome sequencing using Japanese subjects with sporadic schizophrenia. The observed exome point mutation rate in schizophrenia in the present study was similar to those of previous exome sequencing studies^11, 13^. While no genes reported in previous trio-based genetic studies of schizophrenia^7–13^ was detected in our cohort, we identified the *de novo* mutation in *TBL1XR1* (p.Phe10Leu), a gene previously found to be associated with neuropsychiatric conditions. O’Roak *et al*. reported two *de novo* point mutations (p.Leu282Pro and p.Ile397SerfX19) in sporadic cases of ASD^14, 20^. Saitsu and colleagues found a *de novo* point mutation (p.Gly70Asp) in a patient with West syndrome and three missense variants in this gene (p.Ala116Ser, p.Gly405Glu, and p.Asn407Ser) in patients with epilepsy^15^. Deletions on 3q26.32, encompassing the *TBL1XR1* gene, have been reproducibly associated with intellectual disability^21, 22^. These results suggest that mutation of the *TBL1XR1* gene may contribute to a genetic vulnerability to multiple neurodevelopmental psychiatric conditions.

The results we obtained in protein structural and functional analysis suggest potential pathogenic impact of Phe10Leu mutation in TBL1XR1 function. TBL1XR1 is a component of the quaternary corepressor complex composed of N-CoR, SMRT and histone deacetylase 3 (HDAC3), which plays a key role in regulating transcription repression^16, 23–25^. Recent studies demonstrated that TBL1XR1 also plays a critical role in recruiting β-catenin to Wnt target gene promoters for transcriptional activation^17^. These results suggest that TBL1XR1 may act as a molecular switch for transcriptional activation and repression, which may be regulated by its posttranslational modification, such as SUMOylation^26^. The results of our protein structural analysis indicate that Phe10Leu substitution in the NTD of TBL1XR1 decreases structural stability of the NTD, which may influence binding to other components of the corepressor complex. In fact, we observed that Phe10Leu substitution decreases interaction between TBL1XR1 and N-CoR, whereas binding of TBL1XR1 with β-catenin is increased, which may explain observed up-regulation of Wnt/β-catenin-mediated transcriptional activity induced by Phe10Leu substitution.

Accumulating evidences suggest that altered Wnt signaling may be implicated in etiopathophysiologies of neurodevelopmental psychiatric conditions, such as schizophrenia and ASD^14, 27, 28^. Up-regulation of Wnt/β-catenin signaling and dysregulated function of NCoR have been documented in multiple types of cancers^29, 30^. High expression of TBL1XR1 has been reported to be associated with a poor prognosis of colorectal cancer^31^. Consistently, Wnt/β-catenin signaling and the N-CoR/TBL1XR1 complex play critical roles in cell proliferation and differentiation^17, 26, 29^. Thus, it would be of interest to investigate how altered Wnt signaling pathway induced by TBL1XR1 mutations may affect cellular processes in brain development.

The *ABCD4* point mutation (p.lle344Thr) found in the present study was also predicted as damaged by *in silico* functional analysis. ABCD4 is an ABC transporter that has been classified as a member of the D subfamily of peroxisomal ABC transporters, and mutations in ABCD4 cause a new inborn error of vitamin B12 (cobalamin) metabolism^32^. Given that decreased levels of cobalamin have been reported in the frontal cortex in schizophrenia and autism compared with controls^33^, the effect of ABCD4 point mutations in the metabolic processes of cobalamin in brains should be investigated in future studies.

In conclusion, we report nine novel *de novo* non-synonymous SNVs as a result of the whole-exome sequencing of 18 trios. In particular, a *de novo* TBL1XR1 point mutation could alter Wnt/β-catenin signaling activity, supporting the potential involvement of altered Wnt signaling pathway in neurodevelopmental psychiatric disorders. Further studies are required to clarify the involvement of TBL1XR1 mutations in neuropsychiatric conditions.

## Materials and Methods

### Sample collection

We recruited 18 trios of patients with schizophrenia and their unaffected parents from the Tokushima University Hospital, Ehime University Hospital, Kochi University Hospital, and Nagasaki University Hospital in Japan. Patients with schizophrenia (1191 in total, 696 men and 495 women, mean age: 59.5 ± 14.5 years) were independently recruited from Tokushima University Hospital in Japan. Schizophrenia was diagnosed according to Diagnostic and Statistical Manual of Mental Disorders (DSM)-IV criteria by at least two expert psychiatrists based on extensive clinical interviews and a review of medical records (Supplementary Table 1). Non-psychiatric control subjects (1986 in total, 833 men and 1153 women, mean age: 38.6 ± 13.3 years) were selected from volunteers recruited from hospital staff, students, and company employees with no documented history of mental illness or psychiatric problems. All subjects were of Japanese origin. This study was approved by the ethics committees of Ehime, Kochi, Nagasaki, and Tokushima Universities. All enrolled participants provided their signed written informed consent for participation. This study was carried out in accordance with the World Medical Association’s Declaration of Helsinki.

### Exome capture and sequencing

Genomic DNA was extracted from blood leukocytes using the QIAamp DNA Blood Mini Kit (Qiagen, Hilden, Germany). Exome enrichment was performed by the TruSeq DNA Sample Prep Kits and TruSeq Exome Enrichment Kit (Illumina, San Diego, CA, USA). Exome sequencing was performed on a HiSeq1000/1500 (Illumina, San Diego, CA, USA).

### Data processing for identification of *de novo* SNVs

Raw sequencing data for each individual was mapped to the human reference genome (build hg19) by using the Burrows-Wheeler Aligner (BWA v0.5.9)^34^. BWA-generated SAM files were converted into BAM format, sorted and indexed using SAMtools v.0.1.19^35^, then processed by Picard (v1.90) to mark duplicated reads. The BAM-formatted files were further processed using the Genome Analysis Toolkit (GATK, v2.6-4 or v2.6-5) according to the GATK’s best-practice recommendations. In brief, the BAM files were processed with GATK tools (RealignerTargetCreator, IndelRealigner, BaseRecalibrator and PrintReads) to perform local realignment around indels and recalibration of the base quality scores, followed by the data compression with the downstream GATK tool (ReduceReads). When using all of the processed BAM files from 18 trios, multi-sample variant calling with the UnifiedGenotyper tool in GATK (v2.6-5) was done to identify SNV and indel candidates. The resulting Variant Call Format file (VCF, version 4.1) was applied to variant quality score recalibration with the GATK VariantRecalibrator tool. Genomic annotations associated with the variants in the VCF file were added using snpEff (v2.0.5d) with the GRCh37.64 database. According to the GATK’s recommendations for variant filtering, the annotated VCF file was used to extract potential true positive variants on the following values: QD < 2.0, MQ < 40.0, FS > 60.0, HaplotypeScore > 13.0, MQRankSum < −12.5, and ReadPosRankSum < −8.0. After the multi-sample VCF file was split into each of the 18 trios using GATK, the trio-based VCF files generated were used to obtain variants that violated Mendel’s law of segregation in the respective families as candidate *de novo* variants. Of these variants, we extracted *de novo* SNV candidates on the following settings: 1) read depth of coverage at SNV sites were 30 or more in both the proband and parents from a trio of interest, 2) SNVs were not present in the dbSNP v137 database and 3) SNVs were seen in only the proband of interest. Candidate *de novo* non-synonymous SNVs were validated by standard Sanger sequencing on an ABI 3130xl DNA Analyzer. Genotyping of the *TBL1XR1* point mutation [c.30 C > G (p.Phe10Leu)] was performed using a commercially available TaqMan probe with the Applied Biosystems 7500 Fast Real Time PCR System, according to the protocol recommended by the manufacturer (Applied Biosystems, CA, USA).

### *In silico* functional analysis

To predict the effect of *de novo* non-synonymous SNVs on protein function, PolyPhen-2 (http://genetics.bwh.harvard.edu/pph2/)^36^, SIFT (http://sift.jcvi.org/)^37^, and PROVEAN (http://provean.jcvi.org/index.php)^38^ were used.

### Protein structural analysis

Structural models of the N-terminal domain (NTD) of TBL1XR1 were built with homology modeling method by using the Swiss Model^39^. The 2xtc.pdb template was used for building the models. Models contained residues 2–75 of TBL1XR1. Folding energies of the models were calculated with FoldX^40^. Contact area for each residue was calculated by using PDBePISA^41^. Structural illustrations were depicted with Swiss PDB Viewer^42^.

### Plasmids and antibodies

The FLAG-tagged TBL1XR1 expression construct was a gift from Dr. Cun-Yu Wang (University of California, Los Angeles)^17^. The FLAG-tagged mutant TBL1XR1 (TBL1XR1^Phe10Leu^) expression construct was made by a PCR-based mutagenesis protocol^43^. For biochemical experiments, the following antibodies were used: rabbit polyclonal anti-β-catenin antibody (Sigma, Beverly, MA, USA), rabbit polyclonal anti-N-CoR antibody (EMD Millipore, Billerica, MA, USA), as well as mouse monoclonal and rabbit polyclonal anti-FLAG antibodies (Sigma, Beverly, MA, USA).

### Transfection and Wnt/β-catenin activity assays with luciferase reporter system

Human embryonic kidney 293FT cells were maintained in Dulbecco’s modified Eagle’s medium nutrient mixture F-12 (DMEM/F12 (1:1); Gibco BRL, Gaithersburg, MD, USA) containing 10% FBS at 37 °C in a 5% CO2/95% air atmosphere. Twelve-well plates were seeded with 1 × 10^6^ 293FT cells in a medium containing 1% FBS. Transfection of the TOPFlash reporter plasmid, Renilla luciferase cDNA in an SV40 (pRL-SV40, as an internal control) and N-CoR expression construct, together with FLAG-tagged TBL1XR1, TBL1XR1^Phe10Leu^ or an empty (mock) vector, was carried out with Lipofectamine 2000 (Invitrogen, Waltham, MA, USA). Two days after transfection, cells were lysed, and Wnt/β-catenin activity was measured using the Promega Dual Luciferase Reporter Assay System (Promega, Fitchburg, WI, USA) and a FLUOstar Luminometer (BMG Labtech, Ortenberg, Germany).

### Co-immunoprecipitation

293FT cells and HT22 cells transfected with the FLAG-tagged TBL1XR1 or TBL1XR1^Phe10Leu^ expression construct or mock vector were lysed in IP buffers [50 mM Tris-HCl, pH 7.4, 150 mM sodium chloride, 1% NP-40, 0.3% sodium deoxycholate, 0.1% sodium dodecyl sulfate, and protease inhibitor mixture (Roche, Basel, Switzerland)] and [50 mM Tris·HCl, pH 7.4, 150 mM sodium chloride, 0.1% NP-40, and protease inhibitor mixture], respectively. Supernatant fractions obtained after centrifugation at 12,000 g for 15 minutes were incubated with primary antibodies and protein G Plus/Protein A agarose (Calbiochem, Darmstadt, Germany). Immunoprecipitates were analyzed with SDS-PAGE followed by Western blotting after extensive washing. Endogenous β-catenin and N-CoR binding to exogenous TBL1XR1 and TBL1XR1 ^Phe10Leu^ was analyzed by densitometry.

## Electronic supplementary material

Supplementary Figure1

Supplementary Table S1
