# Supplementary material for: De novo non-synonymous TBL1XR1 mutation alters Wnt signaling activity
Source: Sci Rep. 2017 Jun 6;7:2887. doi: 10.1038/s41598-017-02792-z (PMC5460159; doi:10.1038/s41598-017-02792-z)

***De novo* non-synonymous TBL1XR1 mutation alters Wnt signaling activity**

Akira Nishi^1^, Shusuke Numata^1^, Atsushi Tajima^2,3^, Xiaolei Zhu^4^, Koki Ito^4^, Atsushi Saito^4^, Yusuke Kato^5^, Makoto Kinoshita^1^, Shinji Shimodera^6^, Shinji Ono^7^, Shinichiro Ochi^8^, Akira Imamura^7^, Naohiro Kurotaki^7^, Shu-ichi Ueno^8^, Nakao Iwata^9^, Kiyoshi Fukui^5^, Issei Imoto^3^, Atsushi Kamiya^4^, and Tetsuro Ohmori^1^

*^1^ Department of Psychiatry, Institute of Biomedical Sciences, Tokushima University Graduate School, Tokushima, Japan*

*^2^Department of Bioinformatics and Genomics, Graduate School of Medical Sciences, Kanazawa University, Ishikawa, Japan*

*^3^Department of Human Genetics, Institute of Biomedical Sciences, Tokushima University Graduate School, Tokushima, Japan*

*^4^Department of Psychiatry and Behavioral Sciences, Johns Hopkins University School of Medicine, Baltimore, MD, USA.*

*^5^Division of Enzyme Pathophysiology, The Institute for Enzyme Research (KOSOKEN), Tokushima University, Tokushima, Japan*

*^6^Department of Neuropsychiatry, Kochi Medical School, Kochi University, Kochi, Japan*

*^7^Department of Neuropsychiatry, Nagasaki University Graduate School of Biomedical*

*Sciences, Nagasaki, Japan*

*^8^Department of Neuropsychiatry, Ehime University Graduate School of Medicine, Ehime, Japan*

*^9^Department of Psychiatry, School of Medicine, Fujita Health University, Toyoake, Aichi, Japan.*

**Supplementary Figure**


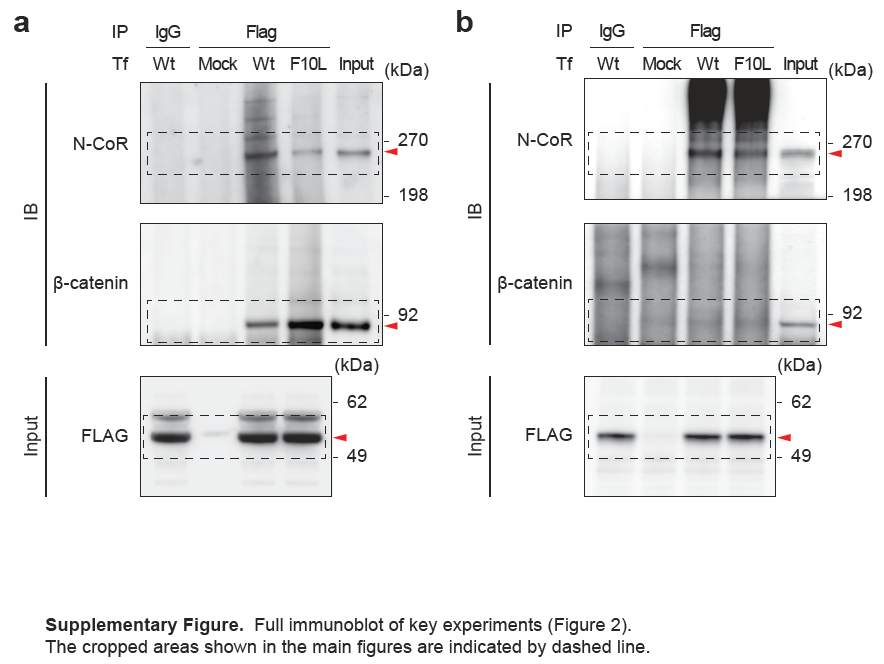

Supplement: Supplementary file 1 — Supplementary Figure1 [file 41598_2017_2792_MOESM1_ESM.doc]
