# Supplementary material for: De novo non-synonymous TBL1XR1 mutation alters Wnt signaling activity
Source: Sci Rep. 2017 Jun 6;7:2887. doi: 10.1038/s41598-017-02792-z (PMC5460159; doi:10.1038/s41598-017-02792-z)
Supplement: Supplementary file 2 — Supplementary Table S1 [file 41598_2017_2792_MOESM2_ESM.doc]

***De novo* non-synonymous TBL1XR1 mutation alters Wnt signaling activity**

Akira Nishi^1^, Shusuke Numata^1^, Atsushi Tajima^2,3^, Xiaolei Zhu^4^, Koki Ito^4^, Atsushi Saito^4^, Yusuke Kato^5^, Makoto Kinoshita^1^, Shinji Shimodera^6^, Shinji Ono^7^, Shinichiro Ochi^8^, Akira Imamura^7^, Naohiro Kurotaki^7^, Shu-ichi Ueno^8^, Nakao Iwata^9^, Kiyoshi Fukui^5^, Issei Imoto^3^, Atsushi Kamiya^4^, and Tetsuro Ohmori^1^

*^1^ Department of Psychiatry, Institute of Biomedical Sciences, Tokushima University Graduate School, Tokushima, Japan*

*^2^Department of Bioinformatics and Genomics, Graduate School of Medical Sciences, Kanazawa University, Ishikawa, Japan*

*^3^Department of Human Genetics, Institute of Biomedical Sciences, Tokushima University Graduate School, Tokushima, Japan*

*^4^Department of Psychiatry and Behavioral Sciences, Johns Hopkins University School of Medicine, Baltimore, MD, USA.*

*^5^Division of Enzyme Pathophysiology, The Institute for Enzyme Research (KOSOKEN), Tokushima University, Tokushima, Japan*

*^6^Department of Neuropsychiatry, Kochi Medical School, Kochi University, Kochi, Japan*

*^7^Department of Neuropsychiatry, Nagasaki University Graduate School of Biomedical*

*Sciences, Nagasaki, Japan*

*^8^Department of Neuropsychiatry, Ehime University Graduate School of Medicine, Ehime, Japan*

*^9^Department of Psychiatry, School of Medicine, Fujita Health University, Toyoake, Aichi, Japan.*

| **Supplementary Table S1** | |  |  |
| --- | --- | --- | --- |
| Summary of samples used in this whole exome sequencing study | |  |  |
| Trio ID | Relationship (Patient=Pt, Father=Fa, Mother=Mo) | Sex (Male=0, Female=1) | Age (y.o.) |
|  |  |  |  |
| trio1 | Pt | 1 | 40 |
|  | Fa | 0 | 76 |
|  | Mo | 1 | 72 |
| trio2 | Pt | 1 | 28 |
|  | Fa | 0 | 56 |
|  | Mo | 1 | 52 |
| trio3 | Pt | 0 | 37 |
|  | Fa | 0 | 69 |
|  | Mo | 1 | 64 |
| trio4 | Pt | 1 | 16 |
|  | Fa | 0 | 55 |
|  | Mo | 1 | 52 |
| trio5 | Pt | 1 | 48 |
|  | Fa | 0 | 79 |
|  | Mo | 1 | 76 |
| trio6 | Pt | 1 | 21 |
|  | Fa | 0 | 49 |
|  | Mo | 1 | 54 |
| trio7 | Pt | 1 | 44 |
|  | Fa | 0 | 75 |
|  | Mo | 1 | 71 |
| trio8 | Pt | 0 | 35 |
|  | Fa | 0 | 74 |
|  | Mo | 1 | 71 |
| trio9 | Pt | 0 | 17 |
|  | Fa | 0 | 54 |
|  | Mo | 1 | 52 |
| trio10 | Pt | 0 | 18 |
|  | Fa | 0 | 56 |
|  | Mo | 1 | 52 |
| trio 11 | Pt | 1 | 36 |
|  | Fa | 0 | 73 |
|  | Mo | 1 | 64 |
| trio 12 | Pt | 1 | 35 |
|  | Fa | 0 | 61 |
|  | Mo | 1 | 62 |
| trio 13 | Pt | 1 | 18 |
|  | Fa | 0 | 47 |
|  | Mo | 1 | 47 |
| trio 14 | Pt | 0 | 17 |
|  | Fa | 0 | 55 |
|  | Mo | 1 | 53 |
| trio 15 | Pt | 0 | 44 |
|  | Fa | 0 | 80 |
|  | Mo | 1 | 72 |
| trio 16 | Pt | 0 | 18 |
|  | Fa | 0 | 45 |
|  | Mo | 1 | 45 |
| trio17 | Pt | 1 | 45 |
|  | Fa | 0 | 77 |
|  | Mo | 1 | 78 |
| trio18 | Pt | 0 | 15 |
|  | Fa | 0 | 46 |
|  | Mo | 1 | 40 |
